# A Lightweight ECC-Based Authentication and Key Agreement Protocol for IoT with Dynamic Authentication Credentials

**DOI:** 10.3390/s24247967

**Published:** 2024-12-13

**Authors:** Momo Li, Shunfang Hu

**Affiliations:** School of Mathematics and Computer Science, Yunnan Minzu University, Kunming 650504, China; 040276@ymu.edu.cn

**Keywords:** internet of things, anonymity, authentication and key agreement, dynamic authenticated credentials, ephemeral information leakage attacks

## Abstract

Due to the openness of communication channels and the sensitivity of the data being collected and transmitted, securing data access and communication in IoT systems requires robust ECC-based authentication and key agreement (AKA) protocols. However, designing an AKA protocol for IoT presents significant challenges, as most IoT sensors are deployed in resource-constrained, unattended environments with limited computational power, connectivity, and storage. To achieve anonymous authentication, existing solutions typically rely on shared temporary public keys to mask device IDs or validate sender certificates, which increases the computational overhead. Furthermore, these protocols often fail to address crucial security concerns, such as nonresistance to ephemeral secret leakage (ESL) attacks and a lack of perfect forward security. To mitigate the computational burden, we propose a dynamic authenticated credentials (DACs) synchronization framework for anonymous authentication. Then, we introduce an ECC-based AKA scheme that employs DACs in place of temporary public keys or sender credentials, enabling efficient and secure anonymous authentication. The security of the proposed protocol was rigorously verified under the Real-or-Oracle model and validated using ProVerif. Performance comparisons demonstrate that our scheme offered significant improvements in security, with an over 37% reduction in communication cost and computational overhead.

## 1. Introduction

**Figure 1:**
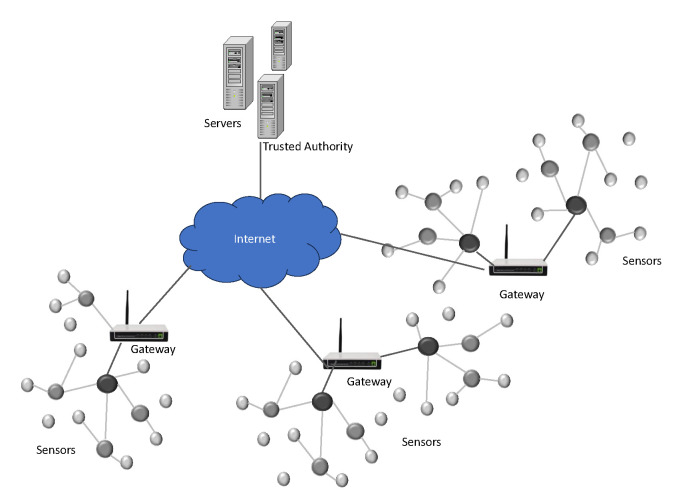
IoT network model.

The Internet of Things (IoT) has revolutionized the way devices, machines, and individuals connect and interact, enabling seamless interoperability regardless of time or location. IoT applications are typically categorized into time-driven and event-driven applications [1]. In event-driven applications, sensors detect specific events, such as the movement or appearance of the target, within a predefined area. Upon detecting such events, the data are transmitted through specialized network architectures to ensure timely communication. In contrast, time-driven IoT applications, as illustrated in Figure 1, involve sensors that periodically collect data, such as temperature, pressure, and humidity, and transmit them to central servers. This process, referred to as periodic data collection [2], is particularly relevant for applications where real-time information is crucial. Given that the data collected by IoT sensors often involve sensitive information tied to personal privacy [3], ensuring robust security is of paramount importance.

Authentication and key agreement (AKA) protocols are central to securing IoT systems, providing key features, such as anonymous authentication, privacy protection, confidentiality, integrity, and non-repudiation. These protocols rely on shared cryptographic keys to protect data. However, IoT systems often face unique security challenges. Many sensors are installed in unattended environments, making them vulnerable to physical attacks. Furthermore, the data they collect are transmitted over wireless channels that are susceptible to interception, modification, and deletion by malicious actors [4,5]. Given the limited computational resources of many IoT devices, there is a pressing need for lightweight AKA protocols that can provide strong security guarantees while minimizing the computational and communication overheads [6].

Several IoT authentication schemes employ lightweight cryptographic techniques, such as symmetric encryption or hashing-based synchronization [7]. However, these approaches can expose systems to various vulnerabilities, including desynchronization attacks [8]. Public-key cryptography, particularly Elliptic Curve Cryptography (ECC), has been identified as a promising solution, offering strong security with smaller key sizes, thus reducing computational, storage, and bandwidth requirements. ECC has been incorporated into AKA protocols for IoT [9], providing a balance between security and efficiency. Nevertheless, designing ECC-based AKA protocols for IoT with a minimal overhead remains a complex challenge.

As shown in Table 1, several ECC-based authentication and key agreement (AKA) schemes have been proposed for IoT in recent years. To ensure anonymity, Tsai et al. [10] used temporary dynamic public keys to encrypt and decrypt the identity information of the sensor. Although this method improves anonymity, it also leads to an increased computational overhead. Similar trade-offs are observed in other schemes [11,12,13,14,15,16,17,18,19,20,21,22,23]. Furthermore, some protocols [23,24,25,26,27,28,29] do not provide sufficient anonymity and remain susceptible to identity leakage under specific conditions. Furthermore, certain schemes [14,18,20,22,23] risk compromising availability while trying to maintain anonymity. For example, when a sensor sends an authentication request or response without including its identity information, or if the protocol is inadequately designed, the server may be required to search through stored data and recompute the temporary public key to identify the sensor. Moreover, an AKA protocol must be resistant to ephemeral secret leakage (ESL) attacks, as ephemeral keys may be precomputed and stored in insecure memory [30]. However, schemes such as [10,11,16,17,18,21,23,24,25,27,31,32] do not effectively mitigate ESL attacks, leaving session keys vulnerable if ephemeral keys are compromised. Finally, perfect forward secrecy (PFS) ensures that even if an attacker gains access to a private key or long-term authentication information, past session keys remain secure and cannot be decrypted. However, existing schemes [11,13,17,24,25,26,28,33,34] do not provide PFS. Additionally, many of these protocols are either prone to other attacks or are not lightweight enough for resource-constrained IoT environments. In conclusion, while various ECC-based AKA schemes enhance the anonymity and security in IoT, many come with significant trade-offs, including an increased computational overhead, vulnerability to ESL attacks, and a lack of PFS. These limitations make them unsuitable for IoT environments that require both robust security and efficiency in resource-constrained settings.

Inspired by fraud detection techniques [35,36] and the provision of perfect forward secrecy (PFS) [37,38] based on dynamic authenticated credentials (DACs), we propose a DAC synchronization framework for anonymous authentication. To ensure feasibility, we modified the DAC update strategy. To maintain synchronization between the server and the sensor, both update the DACs using the accepted session key. The server holds off on deleting the previous DAC until the next one is received from the sensor. This reduces the communication overhead to only two messages, matching the number of communications in the authentication and key negotiation process. If a sensor node uses the same DAC for repeated logins, it will be resynchronized by the server. To address potential inconsistencies in the DAC due to power interruptions or calculation errors, the server can resynchronize any unlogged sensors using a resynchronization procedure.

We then propose an ECC-based AKA scheme that utilizes DACs that reduce overhead and improves security. First, our approach uses DACs for anonymous authentication instead of temporary public keys or sender credentials. Moreover, the proposal provides PFS and resistance to ephemeral secret leakage (ESL) attacks since session keys are derived from both long-term and ephemeral credentials. Even if long-term or ephemeral keys from a single session are compromised, past and future session keys remain secure. Performance evaluations showed that our protocol reduced the communication costs by more than 5% and the computational overhead by more than 37%.

The contributions of this work are as follows:

(1) A literature survey that analyzes efficiency, anonymity, PFS, and vulnerabilities to ESL attacks in related schemes, identifying potential security concerns and weaknesses.
(2) A novel DAC synchronization framework integrated with a lightweight ECC-based anonymous AKA protocol. This framework reduced the computational overhead by more than 37% while improving security features and reducing communication overhead compared with existing schemes.
(3) Formal analysis and automatic verification of the proposal, which guaranteed mutual authentication, anonymity, PFS, and resistance to ESL attacks.

This paper is organized as follows: Section 2 introduces the network model and complexity assumptions. In Section 3, we present the proposed scheme. Following this, in Section 4, Section 5, Section 6 and Section 7, we offer the security analysis results and performance comparison. Finally, in Section 8, we conclude this paper.

## 2. Preliminaries

### 2.1. Network Model

Based on the network models in recent schemes [20,21,22], the network consists of sensor nodes, gateways, and servers. As illustrated in Figure 1, sensor nodes and servers operate in periodic cycles determined by the frequency of data collection and monitoring requirements. Trusted Authorities (TAs) function as specialized entities responsible for key generation, distribution, management, and facilitating secure communication within the system. Both sensors and servers can register with various TAs, enabling them to join different systems. An IoT application may consist of one or more servers, depending on the architecture.

In our proposal, we assumed the involvement of $S_{s}$ (where 1≤s≤m), $SP_{sp}$ (where 1≤sp≤n), and $TA_{l}$ (where 1≤l≤z), where m≫n,m≫z. Here, *m*, *n*, and *z* denote the respective numbers of sensors, servers, and TAs.

The operational cycles of sensor nodes and servers are influenced by factors such as the data collection frequency, environmental monitoring needs, and security requirements. For example, the cycles of the sensor nodes are shaped by the data collection rate, battery life, and the necessity for periodic updates. In contrast, server cycles are determined by the network load and the need to handle authentication processes or update credentials.

### 2.2. Complexity Assumptions

Consider a non-singular elliptic curve *E* defined over a finite field $F_{p}$, where *p* is a large prime. Let *G* be the additive group of points on *E*, with order *q* and base point *P*, where *q* is also a large prime number.

**Definition** **1.** Elliptic Curve Discrete Logarithm (ECDL) Harness Assumption: Given 〈P,xP∈G〉, finding $x\in Z_{q}^{*}$ is computationally difficult. The probability that an algorithm A can solve this problem in time t is negligible for sufficiently small ϵ:

(1) $${\mathrm{Adv}}_{\mathrm{ECDL}}^{A}\left(t\right)=\mathrm{Pr}\left[A\left(P,xP\right)=x:x\in Z_{q}^{*}\right]<\epsilon$$

**Definition** **2.** *Computational Diffie–Hellman (CDH) Hardness Assumption: Given 〈xP,yP∈G〉, computing xyP∈G is computationally hard. The probability that an algorithm A can solve this problem in time t is negligible for sufficiently small ϵ:*

(2) $${\mathrm{Adv}}_{\mathrm{CDH}}^{A}\left(t\right)=\mathrm{Pr}\left[A\left(xP,yP\right)=xyP:x,y\in Z_{q}^{*}\right]<\epsilon$$

### 2.3. Threat Model

The threat model is based on the eCK adversary model [39] and the state-of-the-art schemes [20,21,22]. The adversary A is assumed to possess the following capabilities:

T1. Channel control: A can completely control the communication channels, allowing them to intercept, modify, block, or delete messages exchanged between the server and the sensor.

T2. Session key compromise: A can capture session keys used in previous exchanges between the server and the sensor.

T3. Long-term secret exposure: A can compromise either the sensors or the server, gaining access to their long-term secrets.

T4. Ephemeral secret exposure: A has the ability to obtain ephemeral secrets from both the server and the sensor.

### 2.4. Evaluation Criteria

The evaluation criteria are based on current state-of-the-art schemes [20,21,22]. The criteria include the following:

E1. Mutual authentication and key agreement: The scheme must ensure mutual authentication between the user and the sensor, as well as the successful establishment of a shared session key.

E2. Perfect forward secrecy (PFS): The shared session key must remain secure and unrecoverable, even if the adversary captures the long-term secrets of the server, TA, or sensor.

E3. Anonymity: The scheme should protect the identities of the entities, prevent activity correlation, and ensure untraceability.

E4. Resistance to classic attacks: The protocol must withstand various classic attacks, such as KCI attacks, ephemeral secret leakage, unknown key share, known key, impersonation, desynchronization, replay, and IoT node capture attacks.

## 3. The Proposed Scheme

This section briefly describes the proposal, which includes system initialization and preloading, server and sensor registration, authentication and key agreement, and resynchronization. Table 2 shows some symbols.

### 3.1. System Initialization and Preloading

To select the system parameters, the TA follows these steps:

STA1: The TA chooses an elliptic curve E(a,b) with a base point *P* over $Z_{q}^{*}=\{0,1,\ldots ,q-$1}, where *q* is a large prime number.

STA2: The TA selects two one-way hash functions: $h:{\left\{0,1\right\}}^{*}\to {\left\{0,1\right\}}^{l}$ and $h_{1}:{\left\{0,1\right\}}^{*}\to {\left\{0,1\right\}}^{128}$.

- Function h(·): This hash function is used to securely combine various pieces of information, such as session identifiers, public keys, and other relevant data, into a single value.
- Function $h_{1}\left(\cdot \right)$: This hash function is specifically utilized for the generation and updating of DACs.

STA3: Finally, the TA preloads the tuple $\left\{\left(E\left(a,b\right),q,P,h,h_{1}\right)\right\}$, along with its own identifier, to each sensor and server.

### 3.2. Registration

The registration process for each sensor node S and server SP with the TA is detailed in the following steps:

R1: First, S chooses a random number $r_{s}\in Z_{q}^{*}$. Next, S computes $R_{s}=r_{s}\cdot P$ and securely sends the registration request $\left\{ID_{s},R_{s}\right\}$ to the TA.

Similarly, for server SP, SP selects a random number $r_{sp}\in Z_{q}^{*}$. Then, SP calculates $R_{sp}=r_{sp}\cdot P$ and securely transmits $\left\{ID_{sp},R_{sp}\right\}$ to the TA.

R2: The TA selects a random number $r_{ta}^{s}\in Z_{q}^{*}$ for each valid *S* with ID $ID_{s}$. Then, the TA computes the public key of *S* using $PK_{s}=R_{s}+r_{ta}^{s}\cdot P$ and the DAC of *S* using $TC_{ssp}=h_{1}\left(ID_{s}∥r_{ta}^{s}\right)$.

For server SP, the TA selects a random number $r_{ta}^{sp}\in Z_{q}^{*}$ for each valid $ID_{sp}$ and computes the public key $PK_{sp}=R_{s}+r_{ta}^{sp}\cdot P$. Then, the TA selects another random number $X_{sp}$ for each valid SP.

R3: The TA stores $\left\{ID_{s},PK_{s},ID_{sp},PK_{sp},X_{sp}\right\}$ and then transmits $\{TC_{ssp},PK_{s},r_{ta}^{s},ID_{sp},$$PK_{sp},X_{sp}\}$ to *S* and $\left\{PK_{sp},r_{ta}^{sp},X_{sp},ID_{s},TC_{ssp},PK_{s}\right\}$ to SP through secure channels.

R4: Upon receiving the reply message, *S* calculates the private key $k_{s}=\left(\left(r_{s}+r_{ta}^{s}\right)\;\mathrm{mod}\;\;q\right)$. Then, *S* checks whether $PK_{s}=k_{s}\cdot P$. If valid, S computes $WT_{sp}=k_{s}\cdot PK_{sp}$ and stores $\left\{ID_{s},PK_{s},k_{s},TC_{ssp},ID_{sp},WT_{sp},X_{sp}\right\}$.

Similarly, SP computes the private key $k_{sp}=\left(\left(r_{sp}+r_{ta}^{sp}\right)\;\mathrm{mod}\;\;q\right)$ and computes $WT_{s}=k_{sp}\cdot PK_{s}$. Then, SP initializes the pre-DAC and S-login tokens, and sets $prTC_{ssp}=TC_{ssp}$ and $Login_{s}=Null$. Finally, SP stores $\left\{ID_{sp},PK_{sp},k_{sp},X_{sp},ID_{s},prTC_{ssp},TC_{ssp},Login_{s},WT_{s}\right\}$.

When a new or frozen sensor $S^{\prime }$ is registered, the TA securely transmits $\left\{ID_{s^{\prime }},TC_{s^{\prime }sp},PK_{s^{\prime }}\right\}$ to the corresponding SP.

### 3.3. Authentication and Key Agreement

**Figure 2:**
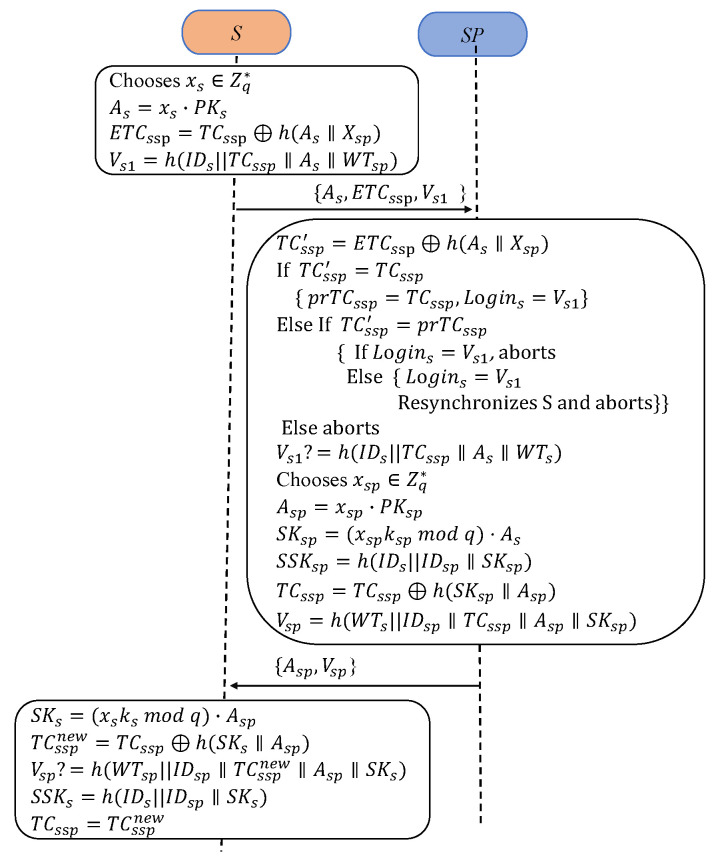
Authentication and key agreement.

As shown in Figure 2, this subsection describes the mutual authentication and key agreement processes between *S* and SP without the help of the TA.

A1: First, sensor *S* selects a random nonce $x_{s}\in Z_{q}^{*}$. Then, *S* computes $A_{s}=x_{s}\cdot PK_{s}$ and $ETC_{ssp}=TC_{ssp}⊕h\left(A_{s}∥X_{sp}\right)$. Next, *S* determines its verifier $V_{s1}=h(ID_{s}∥TC_{ssp}∥A_{s}∥WT_{sp})$. Finally, *S* sends the authentication request message $\left\{A_{s},ETC_{ssp},V_{s1}\right\}$ to the server SP.

A2: In response, SP computes $TC_{ssp}^{\prime }=ETC_{ssp}⊕h\left(A_{s}∥X_{sp}\right)$ to verify the DAC received against $TC_{ssp}^{\prime }$. If $TC_{ssp}^{\prime }=TC_{ssp}$, then $prTC_{ssp}=TC_{ssp}$ and $Login_{s}=V_{s1}$ are set. Else, if $TC_{ssp}^{\prime }=prTC_{ssp}$, SP processes as follows:

If $Login_{s}=V_{s1}$, SP terminates the session.

Otherwise, SP sets $Login_{s}=V_{s1}$, sends a resynchronization message to *S*, and terminates the session.

If neither condition holds, the session is aborted.

Finally, SP confirms the integrity of the incoming message and the validity of *S* by checking whether $V_{s}=h(ID_{s}∥TC_{ssp}∥A_{s}∥WT_{s})$. If invalid, the session is aborted.

A3: First, server SP selects a random nonce $x_{sp}\in Z_{q}^{*}$ and computes $A_{sp}=x_{sp}\cdot PK_{sp}$. Then, SP computes $SK_{sp}=\left(\left(x_{sp}k_{sp}\;\mathrm{mod}\;\;q\right)\cdot A_{s}\right)$, which is used to compute the session key as $SSK_{sp}=h(ID_{s}∥ID_{sp}∥SK_{sp})$. Next, SP computes a new DAC for *S*: $TC_{ssp}=TC_{ssp}⊕h\left(SK_{sp}∥A_{sp}\right)$. After this, SP obtains its verifier $V_{sp}=h(WT_{s}∥ID_{sp}∥TC_{ssp}∥A_{sp}∥SSK_{sp})$. Finally, SP transmits the reply message $\left\{A_{sp},V_{sp}\right\}$ to *S*.

A4: In response, S first computes $SK_{s}=\left(\left(x_{s}k_{s}\;\mathrm{mod}\;\;q\right)\cdot A_{sp}\right)$ and generates a new temporal credential $TC_{ssp}^{new}=TC_{ssp}⊕h\left(SK_{s}∥A_{sp}\right)$. Next, S verifies the equivalence of $V_{sp}=h(WT_{s}∥ID_{sp}∥TC_{ssp}^{new1}|A_{sp}∥SSK_{s})$. If the verification is satisfied, *S* determines the session key using $SSK_{s}=h(ID_{s}∥ID_{sp}∥SK_{s})$ and updates $TC_{ssp}$ with $TC_{ssp}^{new}$.

**Remark** **1.** *When there are storage or computation errors, power-down interruptions, etc., these would cause the $TC_{ssp}$ maintained by S to be inconsistent with either $TC_{ssp}$ or $prTC_{ssp}$ on the server side, which then prevents S from logging into SP. SP will resynchronize the unlogged sensors before the end of the collaboration cycle, following the resynchronization procedures.*

### 3.4. Re-Synchronization

SR1: First, server SP selects a random nonce $y_{s}\in Z_{q}^{*}$ and then computes $TC_{ssp}=prTC_{ssp}=h1\left(ID_{s}∥y_{s}\right)$, $MTC_{ssp}=TC_{ssp}⊕h\left(WT_{s}∥X_{sp}\right)$, and $MV_{sp}=h(ID_{sp}∥ID_{s}∥TC_{ssp}∥WT_{s})$. Finally, SP transmits the resynchronization message $\left\{MTC_{ssp},MV_{sp},Resyn\right\}$ to *S*.

SR2: In response, *S* computes $TC_{ssp}^{new}=MTC_{ssp}⊕h\left(WT_{sp}∥X_{sp}\right)$, and then checks whether $MV_{sp}=h(ID_{sp}∥ID_{s}∥TC_{ssp}^{new}∥WT_{sp})$. If the verification is satisfied, *S* sets $TC_{ssp}=TC_{ssp}^{new}$.

## 4. Formal Security Proof

This section discusses the security of the proposal within the eCK security model [39].

### 4.1. Participants

The proposal involves two participants: a sensor S and a server SP. These participants may have multiple oracles, each participating in separate concurrent executions of the protocol. The oracles for *S* and SP are denoted by $S_{i}$ and ${\mathrm{SP}}_{j}$, where i,j∈Z, and any oracle is represented by I∈S∪SP. Each oracle has a session identifier Sid, which represents the messages it sends and receives. There are three possible states for an oracle:

Accept: The oracle reaches this state after receiving the expected message in the protocol.

Reject: The oracle enters this state if an unexpected or incorrect message is received.

⊥: If the oracle does not respond to input, it transitions to this state.

### 4.2. Adversary

Based on threat models, the adversary A controls the public channels and operates in a probabilistic polynomial-time (PPT) manner. A has the ability to inspect, modify, verify, and insert information into communication between oracles. Furthermore, A can gain knowledge of the secret credentials of the participants and can interact with the oracles $S_{i}$ and ${\mathrm{SP}}_{j}$ using the following queries:

(1) $Execute(SM_{i},SP_{j})$: A can obtain the messages $\left\{A_{s},TC_{ssp},V_{s1}\right\}$ and $\left\{A_{sp},V_{sp}\right\}$ using this query.
(2) $Send(I,m_{I})$: A can send a message $m_{I}$ to oracle *I* and receive a reply from *I* according to the protocol.
(3) ESReveal(I): This query allows A to learn the ephemeral secrets of the oracle *I*.
(4) SKReveal(I): A can reveal the session key owned by oracle *I* using this query.
(5) Corrupt(I): A can obtain long-term confidential information held by the oracle *I*.
(6) h(m): This query allows A to obtain a random hash output for message *m*.
(7) Test(I): This query tests the semantic security of the session key $SSK_{s}$ or $SSK_{sp}$. If the session key has not been established, the query returns ⊥. Otherwise, a private coin *d* is flipped:
  - If d=1, the session key $SSK_{s}$ or $SSK_{sp}$ is returned to A.
  - If d=0, a random key of the same size is returned. The goal of A is to distinguish between real session keys and random numbers.
(8) Expire(I): This query removes the session key held by oracle *I*.

### 4.3. Session Definition

**Fresh:** A session se held by oracle *I* is considered fresh if it and its partner session have not been revealed through queries SK Reveal(I), ESReveal(I), or Corrupt(I) before the session expires with query Expire(I), even if the session has not yet formed.

**Partners:** Two oracles, $S_{i}$ and ${\mathrm{SP}}_{j}$, are considered partners if the following conditions are met: (1) Both oracles reach the accept state. (2) Both oracles are fresh. (3) They authenticate each other and agree on a session key using the same session identifier *Sid*.

**Semantic security:** In the game ${\mathrm{Game}}_{\mathrm{AKA}}\left(I,A\right)$, the security of session keys $SSK_{s}$ or $SSK_{sp}$ is simulated. The adversary A may issue a single Test(I) query, and a bit $d^{\prime }$ is returned if the oracle *I* has reached the accept state and is fresh. Although only one Test(I) query is allowed, A can still make multiple other queries to the oracle *I*. Let Pr[Succ(A)] denote the probability that A succeeds in winning the game ${\mathrm{Game}}_{\mathrm{AKA}}\left(I,A\right)$. The advantage of A in breaching the proposal’s semantic security is defined as

(3) $$\mathrm{Adv}\left(A\right)=\left|2\cdot \mathrm{Pr}\left[d^{\prime }=d\right]-1\right|=\left|2\cdot \mathrm{Pr}\left[\mathrm{Succ}\left(A\right)\right]-1\right|$$

**Definition** **3.** *Under the eCK adversary model, an AKA scheme is considered semantically secure if the advantage of the adversary A,Adv(A), is bounded by a sufficiently small value ϵ, that is, Adv(A)≤ϵ.*

### 4.4. Formal Security Analysis

**Theorem** **1.** *Assume that to breach the semantic security of the proposed scheme, the adversary A can perform at most $q_{e}$ Execute( ) queries, $\left.q_{s}Send(\right)$ queries, and $q_{h}h\left(\right)$ queries within a time limit t. Given that the hash output length is l, the advantage of A is bounded by the following:*

(4) $$\begin{array}{cc} Adv(A) & \leq \frac{\left(q_{h}^{2}+2q_{s}\right)}{2^{l}}+\frac{{\left(q_{s}+q_{e}\right)}^{2}}{2(q-1)}\\ \; & +\left(\frac{3q_{s}}{q-1}+\frac{3q_{s}}{2^{l}}\right)\max\left\{{Adv}_{\mathrm{ECDL}}^{A}\left(t\right),{Adv}_{\mathrm{ECD}}^{A}\left(t\right)\right\} \end{array}$$

**Proof.**  We define a series of six games ${\mathrm{GM}}_{i}$ for i=0,1,…,5 to aid in proving the theorem. When A correctly guesses the bit *d* returned by the Test(I) query, the event $S_{i}$ occurs for each game.

$GM_{0}$: In this game, we simulate an actual attack in the random oracle model (ROR). The advantage in this game is the difference between the probability that the adversary succeeds and 0.5:

(5) $$Adv\left(A\right)=|2Pr\left[S_{0}\right]-1|$$

$GM_{1}$: Here, A can use the $\mathrm{Execute}\left(S_{i},SP_{j}\right)$ query to obtain messages such as $\left\{A_{s},TC_{ssp},V_{s1}\right\}$ and $\left\{A_{sp},V_{sp}\right\}$. Next, A checks the authenticity of the session key $SSK_{s}$ (or $SSK_{sp}$) using SK Reveal(I) and Test(I) queries. The messages exchanged during the authentication process do not provide enough information to infer the session key. Therefore, distinguishing between an actual attack and Game $GM_{1}$ is not possible:

(6) $$Pr\left[S_{1}\right]=Pr\left[S_{0}\right]$$

Three lists are used to track the results of various queries:

- $L_{h}$: The inputs and outputs of the queries h().
- $L_{h}^{A}$: The answers to the h() queries asked by A.
- $L_{E}$: The inputs and outputs of the Execute( ) queries.

${GM}_{2}$: This game simulates that A might forge messages accepted by queries Sen() and h(). Semantic security can only be compromised if A detects collisions and sends a valid message. The probability of collisions in the h() outputs is bounded by the birthday paradox $\frac{q_{h}^{2}}{2^{l+1}}$. Furthermore, the probability of collisions in random numbers is at most $\frac{{\left(q_{s}+q_{e}\right)}^{2}}{2(q-1)}$. As a result, it is impossible to distinguish between $GM_{2}$ and $GM_{1}$ unless the mentioned collisions occur:

(7) $$|Pr\left[S_{2}\right]-Pr\left[S_{1}\right]|\leq \frac{{\left(q_{s}+q_{e}\right)}^{2}}{2(q-1)}+\frac{q_{h}^{2}}{2^{l+1}}$$

$GM_{3}$: In this game, we simulate the case where A is able to guess certain parameters and forge messages, such as $\left\{A_{s},TC_{ssp},V_{s1}\right\}$ and $\left\{A_{sp},V_{sp}\right\}$ without using random oracles. If validation fails, the queries are terminated. The probabilities for $\mathrm{Send}\left(A_{s},TC_{ssp},V_{s1}\right)$ and $\mathrm{Send}\left(A_{sp},V_{sp}\right)$ are each bounded by $\frac{q_{s}}{2}$. With these additional operations, Games $GM_{3}$ and $GM_{2}$ are indistinguishable:

(8) $$|Pr\left[S_{3}\right]-Pr\left[S_{2}\right]|\leq \frac{2q_{s}}{2^{l}}$$

$GM_{4}$: This game models the corruption capability of A.A so that it cannot learn $SSK_{s}$ or $SSK_{sp}$ unless it captures ($x_{s},k_{s}$) or ($x_{sp},k_{sp}$). The adversary can attempt to retrieve $x_{s}$ and $x_{sp}$ by issuing Execute() and h() queries. In the following cases, the adversary may retrieve information about the session keys:

(1) If A corrupts both the oracles $S_{i}$ and $SP_{j}$, the attack succeeds with probability $\frac{2q_{s}}{q-1}$.
(2) If A issues $Corrupt\left(S_{i}\right)$ and $ESReveal\left(SP_{j}\right)$, the probability of success is $\frac{q_{s}}{2^{2}}+$$\frac{q_{s}}{q-1}$.
(3) If A issues $ESReveal\left(S_{i}\right)$ and $Corrupt\left(SP_{j}\right)$, the probability is $\frac{q_{s}}{q-1}+\frac{q_{s^{\prime }}}{2^{2}}$.
(4) If both $ESReveal\left(S_{i}\right)$ and $\mathrm{ESReveal}\left(SP_{j}\right)$ are issued, the success probability is $\frac{2q_{s}}{2}$.

In all these cases, A cannot determine the session keys unless it solves the ECDL or ECD problem within the time limit *t*:

(9) $$|Pr\left[S_{4}\right]-Pr\left[S_{3}\right]|\leq \left(\frac{3q_{s}}{q-1}+\frac{3q_{s}}{2^{l}}\right)\max\left\{{Adv}_{\mathrm{ECDL}}^{A}\left(t\right),{Adv}_{\mathrm{ECD}}^{A}\left(t\right)\right\}$$

$GM_{5}$: Game $GM_{5}$ simulates $GM_{4}$ except that the Test() query is terminated if A issues an $h\left(ID_{s}∥ID_{sp}∥SSK_{s}\right)$ query. The maximum probability for A to retrieve $SSK_{s}$ is $\frac{q_{n}^{2}}{2^{t}}$. Hence, ${\mathrm{SSK}}_{s}$ is $q_{h}^{2}/2^{l}$. Hence,

(10) $$|Pr\left[S_{5}\right]-Pr\left[S_{4}\right]|\leq q_{h}^{2}/2^{l+1}$$

Without the correct input for the h() query, A cannot differentiate between the actual session key and a random one:

(11) $$Pr[S_{5}]=1/2$$

Finally, combining all the probabilities, we conclude that Theorem 1 holds true. □

## 5. Automatic Verification Using ProVerif

ProVerif [40] is a widely recognized tool for verifying the security properties of cryptographic protocols, particularly in the context of secrecy, authentication, and resistance to attacks. It uses pi calculus, a process algebra, and Prolog rules to assess the confidentiality of protocols. ProVerif supports a wide range of cryptographic primitives, including Diffie–Hellman key exchanges, hash functions, and symmetric and asymmetric cryptographic algorithms.

In this section, we formally validate the proposed scheme using ProVerif. Table 3 presents the code for a sensor node during the registration and authentication phases. In this setup, *schst* represents a private communication channel between the sensor *S* and the TA during registration. The public channel *chsp* facilitates communication between the sensor *S* and the server *SP* during authentication. Similarly, each SP and TA has their own corresponding processes. The proposed protocol runs in parallel as ((!Sensor)(!Server)(!TA)).

As shown in Table 4, the results show that A cannot decrypt the authentication process or obtain the long-term, ephemeral, and session keys. The proposal offers anonymity and consistency.

## 6. Descriptive Security Analysis

### 6.1. Perfect Forward Secrecy

For $SSK_{s}=h\left(ID_{s}∥ID_{sp}∥SK_{s}\right)$, where $SK_{s}=\left(\left(x_{s}k_{s}\right)\;\mathrm{mod}\;q\right)\cdot A_{sp}$, if an adversary A has obtained long-term secrets $k_{s},k_{sp},WT_{s},$ and $WT_{sp}$, it cannot access the random numbers $x_{p}$ and $x_{sp}$ for each session. This prevents A from determining $SSK_{s}$, ensuring perfect forward secrecy.

### 6.2. Impersonation Attacks Resistance

We first consider impersonation attacks targeting *S*. In this scenario, the adversary does not have access to long-term secrets $k_{s},k_{sp},TC_{ssp},WT_{s}$, and $WT_{sp}$. Without these secrets, A cannot generate a valid authentication request message $\left\{A_{s},TC_{ssp},V_{s1}\right\}$, where $V_{s1}$ includes $WT_{\mathrm{sp}\;}$. As a result, A cannot impersonate *S*. Similarly, the adversary cannot impersonate SP without the corresponding secrets.

### 6.3. IoT Node Capture Attacks Resistance

While A may capture a sensor, it can only extract long-term credentials, such as $\left\{ID_{s},PK_{s},k_{s},TC_{ssp},ID_{sp},WT_{sp}\right\}$. Since each sensor has unique credentials, the adversary can only compromise the session key between the captured sensor and SP. The session key security remains intact for any uncompromised sensor $S_{s}^{\prime }$.

### 6.4. Key Compromise Impersonation Attack Resistance

Even if A gains access to *S*’s credentials, it cannot impersonate SP or authenticate with *S*. This is because A does not have access to the necessary random numbers $x_{s}$ and $x_{sp}$, which are crucial to the verification process. The credentials for *S* are based on $SSK_{s}=$ $h\left(ID_{s}∥ID_{sp}∥SK_{s}\right)$, where $SK_{s}=\left(\left(x_{s}k_{s}\right)\;\mathrm{mod}\;q\right)\cdot A_{sp}$ and $A_{sp}=x_{sp}\cdot PK_{sp}$.

### 6.5. Desynchronization Attacks Resistance

The scheme uses DACs and random numbers to protect against replay attacks and preserve user anonymity. The server prevents the deletion of prDAC to ensure that both parties share the same DAC, even if the sensor does not receive the authentication response from the server. Since participants do not need to synchronize their clocks, the scheme is resilient to desynchronization attacks.

## 7. Performance Comparison

This section compares the computational and communication costs of the proposed scheme with those of related schemes [19,20,21,22,29], as well as their security and functional attributes.

### 7.1. Computation Cost

Let $T_{pm},T_{pa},T_{h}$, and $T_{sk}$ represent the running times for point multiplication, point addition, hash operations, and symmetric key operations, respectively. The server used for the evaluation is a laptop with an Intel Core i5 2 GHz processor, 16 GB RAM, and macOS 13.4.1. For an elliptic curve with $p=2^{192}$, specifically Curve25519, and a point length of 384 bits, the average running times on this server were 1.258 ms for point multiplication, 0.006 ms for point addition, 0.005 ms for hash operations, and 0.007 ms for symmetric key operations. The sensor node used for testing was a Raspberry Pi 3 Model B+ board that featured an ARMCortex-A53 1.4 GHz processor and 1 GB RAM. Under the same testing conditions, the corresponding running times on the sensor node were 2.225 ms for point multiplication, 0.025 ms for point addition, 0.019 ms for hash operations, and 0.035 ms for symmetric key operations.

As shown in Table 5, the proposed scheme consistently outperformed the related schemes in terms of computational efficiency, with reductions that ranged from approximately 37.98% to 50%.

### 7.2. Communication Cost

Assuming that ID,TS,R,H, and *G* represent an identity, timestamp, random number, hash output, and length of an ECC point, respectively, their corresponding lengths are 128 bits, 32 bits, 256 bits, 256 bits, and 384 bits.

As shown in Table 6, the proposed scheme consistently achieved a notable reduction in communication cost in all comparisons, with reductions that ranged from 5.56% to 38.18% when compared with related schemes.

### 7.3. Performance Comparison

We based our evaluation criteria on the schemes in [19,20,21,22,29]. These criteria are divided into two main categories: ideal attributes and security requirements. The ideal attributes were evaluated from a functional perspective, where we examined whether the scheme possessed the desired features. In contrast, security requirements were evaluated from an attack perspective, which focused on whether the adversary A could successfully break the scheme.

As shown in Table 7, the proposed protocol addressed the security vulnerabilities found in recent schemes, such as the lack of anonymity [29]; the absence of PFS [20]; the poor availability [19,22]; and susceptibility to attacks, such as man-in-the-middle [19,20], ESL [21], desynchronization [21], and DoS [22]. In conclusion, the proposed protocol provided the highest level of security and effectively met the functional and security requirements of IoT environments.

The performance analysis led to the following conclusions: In terms of efficiency, the proposed protocol demonstrated lower computation and communication overheads compared with recent schemes. It consistently outperformed related protocols in computational efficiency, where it achieved reductions that ranged from approximately 37.98% to 50%. In addition, the proposed scheme also achieved significant reductions in communication costs, with improvements that ranged from 5.56% to 38.18% compared with other schemes. Regarding security, our protocol offered comprehensive security properties and effectively mitigated known attacks. It satisfied the security requirements of IoT applications, which ensured robust protection against a wide range of threats.

## 8. Conclusions

We examined existing security schemes and discovered that many use shared temporary public keys to conceal IDs or validate sender certificates, resulting in an increased computational overhead. Additionally, many of these schemes have not covered the necessary security features, such as the lack of PFS and vulnerability to ESL attacks. To overcome these issues, we modified a new DAC framework and developed the proposed protocol with DACs. After the formal analysis and automated verification, we determined that the proposal allowed for mutual authentication, anonymity, and PFS, and was resistant to common attacks, such as ESL, KCI, and MIM. The performance analysis showed that the proposal offered more security features, required fewer communication overheads, and reduced the computation overhead by more than 37%.

## Figures and Tables

**Table 1:** Summary of relevant ECC-based AKA protocols.

Scheme	Year	Shortcomings
[10]	2016	Prone to ESL attacks and high computation cost.
[23]	2016	Prone to DoS and ESL attacks, lacking anonymity, and poor availability.
[11]	2017	Prone to ESL attacks, lacking PFS, and high computation cost.
[33]	2017	Prone to IM attacks, lacking PFS, and high computation cost.
[24]	2017	Prone to ESL and private key leakage attacks and lacking PFS and anonymity.
[25]	2017	Prone to ESL attacks and lacking PFS, anonymity, and mutual authentication.
[12]	2018	Prone to MIM and KCI attacks.
[13]	2018	Prone to MIM and KCI attacks, lacking PFS, and the key control problem.
[14]	2019	Prone to DoS attacks and poor availability.
[26]	2019	Prone to replay and DoS attacks and lacking anonymity and mutual authentication.
[27]	2019	Prone to ESL attacks, lacking anonymity, and high computation cost.
[15]	2020	Prone to KCI attacks and high computation cost.
[31]	2020	Prone to ESL attacks and high computation cost.
[28]	2020	Prone to KCI attacks and lacking PFS and anonymity.
[16]	2021	Prone to KCI and ESL attacks.
[34]	2021	Lacking PFS and high computation cost.
[17]	2021	Prone to MIM, IM, DoS, and ESL attacks and lacking PFS.
[18]	2021	Prone to MIM, IM, and ESL attacks and poor availability.
[29]	2022	Lacking anonymity.
[19]	2022	Prone to MIM attacks.
[20]	2022	Prone to MIM and KCI attacks, lacking PFS, and poor availability.
[32]	2023	Prone to ESL attacks and high computation cost.
[21]	2023	Prone to ESL and desynchronization attacks.
[22]	2024	Prone to DoS attacks, high computation cost, and poor availability.

ESL: ephemeral secret leakage; KCI: key compromise impersonation; MIM: man-in-the-middle; IM: impersonation; PFS: perfect forward secrecy.

**Table 2:** Notations used in the protocols.

Notation	Description
*S*	Sensor node
SP	Server
TA	Trusted third party
A	Adversary A
SSK	Session key
$ID_{i}$	Identity of protocol entity *i*
$r_{i}$,$x_{i}$	Temporary secret of entity *i*
$k_{i}$	Long-term keys of entity *i*
$WT_{i}$	Public key-based signature
$TC_{ssp}$	Dynamic authenticated credential of *S* to SP

**Table 3:** Codes for sensor.

let Sensor =
new rs: bitstring;
let Rs = Mul(rs, P) in
out (schst, (IDs, Rs));
in (schst, (uTCssp: bitstring, uPKs: bitstring, ursta: bitstring, uIDsp: bitstring, uPKsp: bitstring));
let ks = Add (rs, ursta) in
let PKs = Mul (ks, P) in
if PKs = uPKs then
let WTsp = Mul (ks, uPKsp) in
!
(
event startAuthsp;
let As = Mul(xs, PKs) in
let ETCssp = H(Con(uTCssp, Con (As, Xsp))) in let Vs1 = H(Con (Con (Con (IDs, uTCssp), As), WTsp)) in
out (chsp, (As, ETCssp, Vs1));
in (chsp, (sAsp: bitstring, sVsp: bitstring));
let SKs = Mul (Mul (xs, ks), sAsp) in
let TCsspnew = Xor (TCssp, H(Con(SKs, sAsp))) in
let uVsp = H(Con(Con (Con (Con (WTsp, IDsp),
TCsspnew), sAsp), SKs)) in
if uVsp = sVsp then
let ssks = H(con(con(IDs, IDsp), SKs)) in
let TCssp = TCsspnew in event endAuths;
0
).

**Table 4:** Simulation results.

Result	Target
RESULT Weak secret IDs is true (bad not derivable)	Anonymity
RESULT Weak secret IDsp is true (bad not derivable)	Anonymity
RESULT not attacker(ks[]) is true	Long-term secret security
RESULT not attacker(ksp[]) is true	Long-term secret security
RESULT not attacker(xs[]) is true	Ephemeral secret security
RESULT not attacker(xsp[]) is true	Ephemeral secret security
RESULT not attacker (SSKs[]) is true	Session key security
RESULT not attacker (SSKsp[]) is true	Session key security
RESULT inj-event(endAuths) ⇒ inj-event(startAuths) is true	Consistent
RESULT inj-event(endAuthsp) ⇒ inj-event(startAuthsp) is true	Consistent
RESULT inj-event(endAuthsp) ⇒ inj-event(endAuths) is true	Consistent
RESULT inj-event(endAuths) ⇒ inj-event(endAuthsp) is true	Consistent

**Table 5:** Computation costs.

Scheme	Sensor (ms)	Server (ms)	Total (ms)	Decline (in %)
Ours	2$T_{pm}$ + 5$T_{h}$≈7.656	2$T_{pm}$ + 5$T_{h}$≈2.541	10.197	-
[29]	3$T_{pm}$ + 4$T_{h}$≈11.372	4$T_{pm}$ + 2$T_{pa}$ + 5$T_{h}$≈5.069	16.441	37.98%
[19]	4$T_{pm}$ + 4$T_{h}$≈15.12	4$T_{pm}$ + 4$T_{h}$ ≈5.052	20.172	49.45%
[20]	4$T_{pm}$ + 7$T_{h}$≈15.216	4$T_{pm}$ + 7$T_{h}$≈5.067	20.283	49.73%
[21]	3$T_{pm}$ + 4$T_{h}$ + 2$T_{sk}$≈11.49	4$T_{pm}$ + 4$T_{h}$ + 3$T_{sk}$ ≈5.073	16.563	38.44%
[22]	4$T_{pm}$ + 6$T_{h}\;+\;2T_{k}$≈15.312	4$T_{pm}$ + 6$T_{h}\;+\;2T_{k}$≈5.082	20.394	50.00%

**Table 6:** Communication costs.

Scheme	Sensor	Server	Total	Decline (in %)
Ours	G + H + ID	G + H	1088 bits	-
[29]	2G + 2H + TS	G + H	1568 bits	30.61%
[19]	G + H + T + ID	G + H + TS	1152 bits	5.56%
[20]	G + 2H + TS	G + 2H + ID	1376 bits	20.93%
[21]	G + H + R + 2TS + 2ID	G + H + 2TS + ID	1472 bits	26.09%
[22]	2TS + 2H + G + ID	TS + ID + H + G	1760 bits	38.18%

**Table 7:** Performance comparison.

Scheme	F1	F2	F3	F4	F5	F6	F7	F8	F9	F10	F11	F12	F13
[29]	×	✔	✔	✔	✔	✔	✔	✔	✔	✔	✔	✔	✔
[19]	✔	✔	✔	✔	✔	✔	✔	✔	✔	×	✔	✔	✔
[20]	✔	×	✔	×	✔	✔	✔	✔	✔	×	✔	×	✔
[21]	✔	✔	✔	✔	×	✔	×	✔	✔	✔	✔	✔	✔
[22]	✔	✔	✔	×	✔	×	✔	✔	✔	✔	✔	✔	✔
Ours	✔	✔	✔	✔	✔	✔	✔	✔	✔	✔	✔	✔	✔

F1: anonymity; F2: PFS; F3: mutual authentication without RC/TA; F4: availability; F5: ESL resistance; F6: DoS attack resistance; F7: desynchronization attack resistance; F8: IoT node capture attacks resistance; F9: impersonation attack resistance; F10: man-in-the-middle attack resistance; F11: replay attack resistance; F12: key comprise impersonation attack resistance; F13: unknown key share attack resistance; ✔: a functional feature or security is supported by the scheme; ×: a feature is not supported or a scheme is insecure.

## Data Availability

The raw data supporting the conclusions of this article will be made available by the authors on request.
